# Supplementary figures and images for: Threshold of increase in oxygen demand to predict mechanical ventilation use in novel coronavirus disease 2019: A retrospective cohort study incorporating restricted cubic spline regression
Source: PLoS One. 2022 Jul 14;17(7):e0269876. doi: 10.1371/journal.pone.0269876 (PMC9282654; doi:10.1371/journal.pone.0269876)

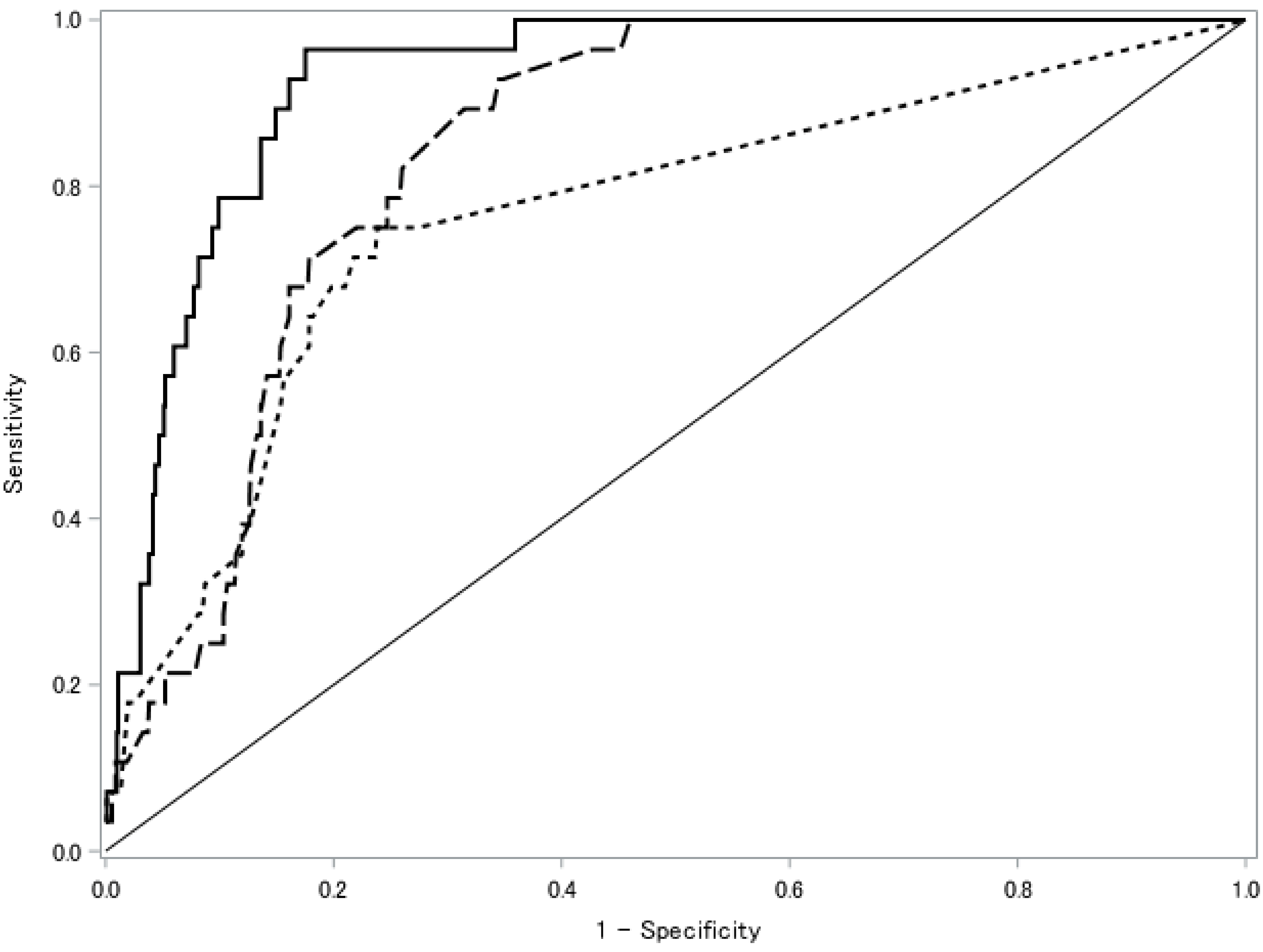

Supplement: S1 Fig — Sensitivity analysis was conducted by excluding negative changes in oxygen demand. Increments in oxygen demand to predict mechanical ventilation use within 12 h was evaluated by ROCs in several models as follows: simple model only using increments in oxygen demand (AUC 0.774 [0.689–0.860]); combination model using both amounts and increments in oxygen demand (AUC 0.873 [0.834–0.911]); and a fully adjusted model including amounts and increments in oxygen demand, RR, Ct value of PCR for SARS-CoV-2, and days from positive PCR (0.927 [0.897–0.957]). Abbreviations: ROC, Receiver operating curve; AUC, area under the ROC; RR, respiratory rate; Ct, quantification cycle; PCR, polymerase chain reaction; and SARS-CoV-2, severe acute respiratory syndrome coronavirus 2. (TIFF) [file pone.0269876.s001.tiff]
