# Supplementary material for: Threshold of increase in oxygen demand to predict mechanical ventilation use in novel coronavirus disease 2019: A retrospective cohort study incorporating restricted cubic spline regression
Source: PLoS One. 2022 Jul 14;17(7):e0269876. doi: 10.1371/journal.pone.0269876 (PMC9282654; doi:10.1371/journal.pone.0269876)
Supplement: S1 Table — (DOCX) [file pone.0269876.s002.docx]

|  | High increment of oxygen^a^ | Low increment of oxygen | p value | OR/Coefficient (95% CI) | |  |
| --- | --- | --- | --- | --- | --- | --- |
| 90-day mortality, *n (%)* | 67 (42.4%) | 233 (18.6%) | 0.025 | 1.68 | (1.07 to 2.65) |  |
| ICU-free days to day 30, *days, median (IQR)* | 25 (15–30) | 30 (26–30) | 0.230 | 0.7 | (−0.4 to 1.8) |  |
| Ventilator-free days to day 30, *days, median* (*IQR*) | 30 (30–30) | 30 (30–30) | 0.112 | −0.7 | (−1.5 to 0.2) |  |
| OR = odds ratio, CI = confidence interval, and ICU = intensive care unit. ^a^High increment was defined as higher than the threshold (0.44 L/min/h) obtained from the spline curve (Fig 3). | | | | | |  |
|  |  |  |  |  |  |  |
