# Supplementary material for: Threshold of increase in oxygen demand to predict mechanical ventilation use in novel coronavirus disease 2019: A retrospective cohort study incorporating restricted cubic spline regression
Source: PLoS One. 2022 Jul 14;17(7):e0269876. doi: 10.1371/journal.pone.0269876 (PMC9282654; doi:10.1371/journal.pone.0269876)
Supplement: Attachment — Submitted filename: DonnerOxyIntubate_response_RY050122.docx [file pone.0269876.s003.docx]

**Threshold of Increase in Oxygen Demand to Predict Mechanical Ventilation Use in Novel Coronavirus Disease 2019: A Retrospective Cohort Study Incorporating Restricted Cubic Spline Regression**

**Editor**

**Author Response:** We are grateful for your insightful comments and the reviewers’ valuable suggestions on our manuscript. We have conducted additional analyses and revised the manuscript following comments.

**Reviewer 1**

**Author Response:** We are grateful for your comments on our manuscript. Please see our specific responses below.

1. Please include a separate ethical statement in your methods section, that clearly highlights the consent measures used, and the ethical procedures followed.

**Author Response**: Thank you for pointing this out to us. We added the separated section “Ethical statement” and described the consent measures with ethical procedure in page8 at lines 127-131.

2. In your conclusion segment, I believe it will give your findings more impact if you can phrase them in terms of public and global health impact.

**Author Response**: Thank you for bringing this to our attention. We added sentences in Conclusions to clarify the possible effect by the current study on global health as follows:

In lines 379-380 in Conclusions, “*The threshold identified in this study would be useful for appropriately allocating patients to ICU in regions where resources are overwhelmed due to pandemic of COVID-19*”

**Reviewer 2**

**Author Response:** We are grateful for your comments on our manuscript. Please see our specific responses below.

1. the limited study sample (66 patients included// 11 patients intubated) and relatively early intubation decision (oxygen flow requirements 6-8L) is a problem in terms of extracting wide conclusions from the work.

**Author Response**: Thank you for pointing this out to us. We agree with your thought that the limited sample size and early intubation strategy would limit the generalizability of our study. To clarify this limitation, we added the sentences in Conclusions as follows:

In lines 381-382 in Conclusions, “*the generalizability of threshold should be validated by a multi-center trial with large sample size.*”

2. The list of comorbidities does not include hypertension or diabetes. These conditions may place a patient with covid-19 at a higher risk of severe illness.

**Author Response**: Thank you. We additionally obtained these data and conducted additional analyses. The results were added in Table 1. In addition, words were added in line 159 in Material and methods.

3. The definition of “severe comorbidity” (row 128-129) was not clear. Are these patients included in the study?

**Author Response:** Thank you for bringing this to our attention. The lack of clear description was an oversight in the original manuscript. The severe comorbidity included congestive heart failure requiring oxygen and acute kidney injury requiring hemodialysis. Patients with severe comorbidity were included in the study if they met all the inclusion criteria as other patients. Sentences in the manuscript have been revised to the following:

In lines 123-124 in Materials and methods, “Patients with severe comorbidity, *such as congestive heart failure requiring oxygen and acute kidney injury requiring hemodialysis, were admitted to the ICU*”

4. Low molecular weight heparin (LMWH) is not included in the list of administrated medications. Patients with covid 19 may trigger a coagulopathy state and affect changes in oxygen demand.

**Author Response**: We are grateful for your comments. We additionally obtained the data on unfractionated and low-molecular-weight heparin and identified no patients were treated with low-molecular-weight heparin (probably because it was not covered by insurance). As several patients were treated with unfractionated heparin, we conducted additional analyses. We added this information in Tables 2 and 3, and words were added in lines 164 in Material and methods and 248 and 250-251 in Results.

**Reviewer 3**

**Author Response:** We are grateful for your comments on our manuscript. Please see our specific responses below.

1. Definitely, you need more numbers to empower the results and more than one center for sake of generalizability.

**Author Response**: Thank you for pointing this out to us. We agree with your thought that the limited sample size limited the generalizability of our study. To clarify this limitation, we added the sentences in Conclusions as follows:

In lines 381-382 in Conclusions, “*the generalizability of threshold should be validated by a multi-center trial with large sample size.*”

**Reviewer 4**

**Author Response:** We appreciate your review and comments. Thank you.
